# Construction Notes

**Published:** 1895-10-26

**Authors:** 


### CONSTRUCTION NOTES.

**New Eye, Ear, and Throat Hospital, Cork.**

This new structure, when completed, will be handsome and commodious, and will stand in the forefront of the buildings in the city. It will be faced with Ruabon brick, and ornately dressed with limestone plinth, bands, sills, and cornices. The hospital is planned to consist of three storeys, to be entered through a doorway, porch, and hall, ten feet wide, facing the Western Road; the hall opens into a corridor, connecting day rooms for intern patients, with private wards at the back of the same. There are also on this floor doctor's-room, waiting-room, house surgeon's-room, and porter's-room. To the rear of the main building is situated a waiting-room for the extern department, which will accommodate from 80 to 100 patients. The examination-rooms are approached from the waiting-room, and the usual adjuncts, *i.e.*, dark-room, lavatories, dispensaries, &c., are provided. The kitchen, scullery, &c., is situated in the space between the main building and the extern department. On the first floor there are two large public wards, one at each end, well lighted and ventilated, measuring 36 feet long by 22 feet wide, and connected by a main corridor. Opening from the latter are bath-rooms and water closets, disconnected by means of a ventilating lobby. On the first floor are situated apartments for the matron and the house surgeon, and service-rooms from the kitchen, connected with the latter by means of a hand lift. Over the extern department is an operating-room, two operation bed-rooms, and the usual servants' apartments. The top floor is a repetition of the middle floor, with the addition of an isolation ward, where patients can be completely separated from the rest of the institution. The upper floors throughout will be absolutely fire-proof, having oak blocks laid under foot. The main staircase will also be impervious to heat. The grounds surrounding the institution will be well laid out, the whole area being enclosed with a wall. The architect is Mr. James F. M'Mullen, C.E., of Cork.

### Helensburgh New Infirmary.

The new infirmary at Helensburgh for non-infectious cases has now been opened. This consists of a central pavilion of two storeys, with high-pitched roof, flanked by two octagonal oriels, with bell-shaped roof. The main wards, male and female, of one storey, running east and west, contrast well with the picturesque skyline of the central block. Each main ward contains six beds, and adjacent to the main wards are two private wards, giving accommodation for two beds; while two front rooms are of sufficient size to be available for private wards, should need arise. The floors of the wards, corridors, &c., are stained and varnished throughout. The upper floor of the central part provides sleeping accommodation for the staff. To the rear, and at right angles to the main building, is the kitchen wing, and beyond the kitchen, are placed the washhouse and laundry, coal, and disinfecting chamber, the latter entirely shut off, and entered from the outside only. The mortuary occupies an isolated block, and, like the kitchen wing, has the walls inside lined with glazed bricks. The sanitary arrangements and fittings are of the most improved description. The building is heated by means of hot-water pipes, so arranged in the wards as to warm the fresh air as it enters. The whole work has been carried out from the designs and under the supervision of William Leiper, A.R.S.A., of Glasgow.

### The Devon and Exeter Hospital: A New Wing.

For some years past the accommodation at the Devon and Exeter Hospital has been found inadequate to the demands made upon it, and the committee, with the consent of the governors, have formulated a scheme for enlarging the building. This will be done by erecting a new wing attached to the right-hand side of the main building. The ceremony of laying the foundation-stone has been performed, when a large and representative gathering took place in the grounds of the hospital. The architect is Mr. Charles Cole. A special feature will be made of the sanitary work, which is to be carried out on the latest principles. The main walls will be of brickwork 18 inches in thickness throughout, and the roofs are to be covered with special slates. The wing will be three storeys high, and consist of three main wards, each containing 24 beds, and three small single wards. The main wards are to be 97 ft. long and 26 ft. 6 in. wide, and each will be warmed by means of two central stoves (the vertical flues of which will be swept from the ground level) and eight hot-water coils. Each patient has 1,500 cubic feet of air space. The floors are to be formed of steel joists, with a concrete and terra-cotta lintel, on which will be laid teak floor boards. At the further end of the wing will be a balcony to each floor, and a fire escape staircase. The space underneath the wards will form a covered airing ground. Each floor has a linen-room, kitchen, pantry, &c., and in the sanitary towers are bath-rooms, lavatories, &c., and the walls of which are to be lined with white glazed bricks inside. A lift will be provided, running from the basement to the top floor, for patients and dinner wagons. The present wooden staircase will be removed, and one of iron and concrete constructed in its place, connecting the main building with the wing. The building contract is being carried out by Messrs. Tree and Holley.

